# SARS-CoV-2 infections in professional orchestra and choir musicians—a prospective cohort study

**DOI:** 10.1007/s10654-022-00917-x

**Published:** 2022-09-29

**Authors:** Anne Berghöfer, Gabriele Rotter, Joachim Pankert, Katja Icke, Stephanie Roll, Ryan King, Stefan N. Willich

**Affiliations:** Institute of Social Medicine, Epidemiology and Health Economics, Charité–Universitätsmedizin Berlin, corporate member of Freie Universität Berlin and Humboldt-Universität zu Berlin, Berlin, Germany

**Keywords:** Incidence, Coronavirus SARS, Occupational health, Cohort study

## Abstract

During the COVID-19 pandemic, rehearsal and concert activities of professional orchestras and choirs were severely restricted based on the assumption of particularly high infection risks associated with wind instruments and singing. Therefore, our primary objective was to determine the incidence of SARS-CoV-2 infections in orchestra and choir musicians compared to controls. We also assessed influenza, flu, upper respiratory tract infections, and course of illness. Musicians from professional orchestras and choirs and controls from 23 institutions throughout Germany were included in a prospective cohort study. Data were collected from October 2020 to June 2021 by weekly online surveys. A mixed-effects cox proportional hazards model was used to assess the effect of exposure by professional activity on SARS-CoV-2 infection. In 1,097 participants (46.7 years (SD 10.3); 46.8% female; 705 orchestra, 154 choir, and 238 control subjects) 40 SARS-CoV-2 infections occurred. Cases per person-years were 0.06 in orchestras, 0.11 in choirs, and 0.03 in controls. Hazard ratios compared to controls were 1.74 (95% CI 0.58 to 5.25, *p* = 0.320) for orchestra musicians and 2.97 (0.87 to 10.28, *p* = 0.087) for choir singers. Infection source was suspected predominantly in private contexts. Disease courses were mild to moderate. Other respiratory infections were reported in 6.1% of study weeks in orchestras, 10.1% in choirs, and 8.0% in controls. Sick leave days of total study days were 0.5, 2.1 and 1.3%, respectively. This epidemiologic study during the pandemic in professional musicians indicates no increased risk of SARS-CoV-2 infections in orchestra musicians and a trend towards increased risk in choir singers compared to controls. However, the exact routes of infection could not be validated. If appropriate hygiene concepts are adhered to, safe orchestra and choir activity appears possible in pandemic times.

**Supplementary Information:**

The online version contains supplementary material available at 10.1007/s10654-022-00917-x.

## Introduction

The spread of severe acute respiratory syndrome coronavirus 2 (SARS-CoV-2) beginning in December 2019 rapidly reached the dimensions of a global pandemic. Transmission occurs primarily through aerosols and droplets in the respiratory air, with possible transmission through contact with infectious surfaces or other routes of transmission, including fomite transmission playing a minor role [1]. The size of the particles differs depending on their origin: large particles up to 500 µm originate from the nasal and oral cavities mainly during phonation, coughing, or sneezing, whereas particles of about 1–3 µm are generated in the lower respiratory tract by breathing and in the larynx through phonation [2]. The half-life of aerosol concentration in indoor air is highly dependent on ventilation, ranging from 30 s in well-ventilated (mechanical ventilation and window opening) to 14 min in poorly ventilated rooms [3].

Measures to contain infection led to severe restrictions on public life and individual freedom, including mobility restrictions, in many countries worldwide. Cultural institutions were particularly affected by these restrictions. Rehearsal and concert activities of orchestras and choirs were largely suspended as of spring 2020. The orchestra as a workplace was considered to have a higher risk of infection because of the difficult implementation of minimum distance rules and because woodwind and brass players in the orchestra produce droplets and aerosols that are considered potentially infectious. The choir as a workplace has also been attributed by expert opinions a particularly high risk of infection because singing produces a higher than average amount of droplets and aerosols [4, 5]. The level of particle emission during singing was reported to be 4.0 to 99.5 times that of speaking, and 15.3 to 330 times that of breathing. Also, higher sound pressure levels or pronounced articulation increased the emission of particles [6, 7]. It has been observed that choral rehearsals are a high exposure factor, at least when ventilation is insufficient. These considerations have been supported by reports of high infection rates after choral events [8]. In subsequent studies with wind instruments, aerosol visualizations revealed a maximum dispersion of exhaled air from 20 cm for the flute to 112 cm for the piccolo (when exhaled air passes over the mouthpiece) [9]. Quantitative airflow measurements with sensors at distances of 1, 1.5 and 2 m during playing showed airflow velocities < 0.1 m/s for all musical instruments tested except the tuba (0.13 m/s at 1 m distance) and oboe (0.15 m/s at 1 m and 0.12 m/s at 1.5 m distance). Additional sideward measurements at a distance of 0.5 m resulted in 0.15 m/s for the alto flute and 0.13 m/s for the piccolo [10]. Overall, the results of previous studies from airflow research indicate a much lower exposure to aerosols than originally assumed [11–15]. The scientific evidence on the risk of infection during ongoing performance activities remains sparse and is mainly based on airflow measurements of wind instruments, aerosol visualizations in players and singers, and individual reports of infections after choir rehearsals. From September 2020 to July 2021, an exploratory study at the Bavarian State Opera in Munich used elaborated hygiene management approach and found artistic work at and reopening feasible with a well-controlled risk [16]. Epidemiological studies on the occurrence of SARS-CoV-2 infections under real pandemic conditions during musical activities are so far lacking. As some SARS-CoV-2 infections might persist undetected by antigen [17] or PCR [18] tests, and other potentially airborne infections are also relevant to music performance, the number of sick leave days and the course of illness might be associated with SARS-CoV-2 infection. Findings from these could also play a role after the pandemic.

The primary objective of the present study was, therefore, to determine the incidence of SARS-CoV-2 infections in musicians of professional orchestras and singers of professional choirs, respectively, as compared with non-musician control subjects. Secondary objectives included assessing the incidence of influenza, flu, or upper respiratory tract infection, the number of sick leave days, and the course of illness associated with SARS-CoV-2 infection.

## Methods

### Study design and setting

Musician members of professional concert or opera orchestras and professional concert or opera choirs, as well as control subjects, were recruited from participating institutions throughout Germany in a three-arm prospective cohort study. Data collection took place from October 1, 2020, to June 30, 2021, thus mostly in line with the 2020/21 performance season.

Data was collected prospectively via online survey that could be completed via smartphone, tablet, or personal computer. Participants received letters with information about the study and invitations to participate in both German and English. Consent to study participation was given via a link to the online consent form. After giving their consent, the participants received a baseline questionnaire and subsequently follow-up questionnaires at weekly intervals for up to 9 months. To ensure data protection, questionnaires could only be opened via a personalized link that participants received individually by email.

Exposure was defined as working as a professional orchestral musician or choir singer. Exposure was a proxy variable for individuals being exposed to potentially infectious aerosols and droplets generated by, among other things, wind instruments or singing during regular rehearsals and concert performances in professional orchestras or choirs.

The control group included all individuals not exposed to potentially infectious aerosols or droplets caused by orchestral playing or singing. The primary endpoint was the number of SARS-CoV-2 infections confirmed by positive testing during the study period. Secondary endpoints were the incidence of influenza, flu, or other upper respiratory tract infection, and the number of days of sick leave.

### Participants

All participants had to be at least 18 years old and consent to study participation. Orchestra musicians had to be members of a professional concert or opera orchestra, and singers had to be members of a professional concert or opera choir. Control subjects had to be non-musician employees of the same participating institutions as those of the exposed subjects, and not present in the room during the rehearsal and concert activities of the orchestral and choir musicians. This included various professional groups from administration with no regular contact to the musicians during rehearsal and concert activities but mostly office work. The stage personnel had direct contact with the musicians but regularly not during rehearsal and concert activities. Exclusion criteria were the existence of known infection with SARS-CoV-2 at study inclusion, activity in a string only orchestra, or activity as music student or temporary employee.

### Variables, data collection, and risk score assessment

At baseline, sociodemographic data (age, sex, number of adults and children in the household, occupation including instrument or voice specialty), and health-specific variables being risk factors for a severe course of the illness, height, weight, e.g. chronic diseases, smoking status and also vaccinations status were obtained [19–21].

Subsequently for up to 38 weeks, the occurrence of symptoms of SARS-CoV-2 infection, influenza, flu, or upper respiratory tract infection were collected on a weekly basis for each of the last 7 days. Furthermore, results of testing for SARS-CoV-2 infection (if testing was done), the number of days absent due to illness, and vaccinations given for SARS-CoV-2, influenza, or pneumococcus were recorded. Participants reported the frequency and type of private social contacts and the use of general protective measures in everyday life and at work, as well as specific protective measures of individual musician groups. Furthermore, the length, frequency, location, and sequence of music-making in rehearsals and concerts, possible teaching activities, and tours were assessed. Study participants who tested positive for SARS-CoV-2 were subsequently contacted via email and telephone to obtain information on the suspected infection source and the clinical course of illness, graded following the National Institutes of Health [22].

The highly variable exposure due to the pandemic containment measures was accounted for by the calculation of a weekly professional exposure risk score combining all infection risks of the professional activity, including location, sequence of music-making in rehearsals and concerts, application of general protective measures at the workplace, specific protective measures of individual groups of musicians, possible teaching activities, touring, as well as hygiene concepts at the workplace (room size, ventilation, ensemble set-up, audience concepts, etc.). This score was weighted by each subject’s weekly rehearsal and/or concert time.

A weekly private risk score (range 0 to 28 points) was calculated from the recorded possible confounders, and consists of two parts: 1) confirmed contacts with SARS-CoV-2 positive individuals (contact risk), and 2) other potential risks from the private environment or public space (general risk), including data on vaccinations, frequency and type of contacts with persons at increased risk for SARS-CoV-2 infection, contacts without mouth-nose protection with others, household size and regular contacts with daycare or school children, health care workers and professional teachers, use of public or other transportation, and other general personal risk behavior related to SARS-CoV-2 infection risks. Calculation of the professional exposure risk score and the private risk score and references for the rationales apart from our expert consensus, partly based on former risk calculations [23], are given in detail in the supplementary material table S1.

In addition, the respective hygiene concepts and modalities of performance in the participating ensembles were collected.

All data from the questionnaires were recorded pseudonymously.

### Statistical analysis

For each study group and in total we calculated baseline characteristics for categorical (n; %) and continuous (mean; SD) variables, the cumulative incidence and incidence rate (per year) of SARS-CoV-2 infection, as well as the time at risk (in weeks and years). Cumulative incidence curves were calculated (1 minus survival function) of SARS-CoV-2 infections per study group. The weekly mean of the private and professional exposure risk scores per study group was calculated, as was the weekly proportion of participants with influenza, flu, or other respiratory symptoms and sick leave.

A mixed effects cox proportional hazards model was used to model the effect of the exposure on SARS-CoV-2 infection. The time scale was calendar time and the fixed effects in the model were the exposure group (orchestra, choir, controls) and the mean private risk score (mean score over the entire study period for each subject). The ensembles were considered to be random effects. As a post-hoc secondary analysis, the crude overall exposure (orchestra, choir, control) was stratified by exposure intensity; the orchestra/choir exposures were split into high or low intensity groups based on their respective medians of the mean professional exposure risk score (higher/lower than 22.3 for orchestral musicians, higher/lower than 19.6 for choral singers).

The contact risk has a potentially large confounding effect and adjusting for it helps differentiate between SARS-CoV-2 infections caused by the exposure (orchestra, choir) and infections from non-exposure sources (private contacts). Therefore, to test how sensitive the results are to changes in the contact risk, we included additional information into the contact risk from a follow-up survey performed with SARS-CoV-2 positive individuals that provided information on the suspected source of infection. In further sensitivity analyses, we adjusted either for the contact risk only, for the general risk only, or for both risks separately (two separate variables). All sensitivity analyses used a mixed effects cox proportional hazard model as described above.

The incidence of influenza, flu, or upper respiratory tract infection and the number of sick leave days were modelled using a linear mixed effects model (same fixed/random effects as described above). The respective outcome was defined as the percentage of weeks (with respect to individual study participation time in weeks) that a subject reported influenza, flu, or upper respiratory tract infection as well as the percentage of days that a subject reported sick leave.

All statistical analysis was performed using the R software version 4.1.1. [24].

### Participant and public involvement

As part of stakeholder involvement, the seven Berlin concert and opera orchestras (orchestra boards, artistic directors) and the radio orchestras and their choirs were consulted in the planning and development of the study. They helped design the survey instruments and participated in recruiting participants.

## Results

A total of 1,133 subjects from 23 ensembles gave informed consent to participate in the study, of these 1,120 were included (723 musicians from 17 orchestras, 157 singers from 6 choirs, and 240 control subjects from 22 of the 23 participating ensembles). Twenty three individuals dropped out after baseline and before the first follow-up, thus 1,097 individuals were included into the main analyses.

All instrumental groups were represented among the orchestral musicians, and all voice registers were represented among the singers (Table 1). The control group included various professional groups from administration (n = 41, 17.1%) and stage personnel (n = 199, 82.9%) (Table 1).

**Table 1:** Baseline characteristics by study group and in total. (SD standard deviation)

	Total N = 1,120	Orchestra n = 723	Choir n = 157	Controls n = 240
Age, years, mean *(SD)*	46.7 (10.4)	47.4 (10.0)	48.4 (9.2)	43.6 (11.5)
*Sex n (%)*				
Female	520 (46.4)	265 (36.7)	104 (66.2)	151 (62.9)
Male	598 (53.4)	458 (63.3)	53 (33.8)	87 (36.3)
Diverse	2 (0.2)	0 (0)	0 (0)	2 (0.8)
*Household n (%)*				
Single	179 (16.0)	87 (12.0)	35 (22.3)	57 (23.8)
Multi-person	938 (83.8)	634 (87.7)	121 (77.1)	183 (76.3)
Not specified	3 (0.3)	2 (0.3)	1 (0.6)	0 (0)
Number of children in household *mean (SD)*	1.02 (1.12)	1.11 (1.17)	0.91 (0.99)	0.79 (1.0)
*Instrument n (%)*				
Violin, viola	–	276 (38.2)	–	–
Violoncello, double bass	–	127 (17.6)	–	–
Plucked string instrument	–	7 (1.0)	–	–
Wind instrument	–	136 (18.8)	–	–
Brass instrument	–	131 (18.1)	–	–
Percussion	–	43 (5.9)	–	–
Not specified	–	3 (0.4)	–	–
*Voice category n (%)*				
Soprano	–	–	51 (32.5)	–
Alto	–	–	49 (31.2)	–
Tenor	–	–	23 (14.6)	–
Baritone	–	–	26 (16.6)	–
Bass	–	–	3 (1.9)	–
Not specified	–	–	5 (3.2)	–
*Working area n (%)*				
Stage staff	–	–	–	41 (17.1)
Administration	–	–	–	199 (82.9)
*Vaccinations n (%)*				
Influenza	181 (16.2)	94 (13.0)	33 (21.0)	54 (22.5)
Pneumococcus	72 (6.4)	44 (6.1)	10 (6.4)	18 (7.5)
Body-Mass-Index *mean (SD)*	24.1 (4.2)	23.9 (3.4)	25.4 (5.4)	24.0 (5.3)
*Smoking n (%)*				
Never	704 (62.9)	461 (63.8)	108 (68.8)	135 (56.3)
Currently smoking	130 (11.6)	80 (11.1)	10 (6.4)	40 (16.7)
Formerly smoking	283 (25.3)	179 (24.8)	39 (24.8)	65 (27.1)
Not specified	3 (0.3)	3 (0.4)	0 (0)	0 (0)
*Chronic disease n (%)*				
None	818 (73.0)	535 (74.0)	106 (67.5)	177 (73.8)
Arterial hypertension	103 (9.2)	62 (8.6)	18 (11.5)	23 (9.6)
Cardiac disease	26 (2.3)	17 (2.4)	3 (1.9)	6 (2.5)
Chronic bronchitis	4 (0.4)	2 (0.3)	1 (0.6)	1 (0.4)
Bronchial asthma	49 (4.4)	32 (4.4)	5 (3.2)	12 (5.0)
Other lung disease	8 (0.7)	4 (0.6)	2 (1.3)	2 (0.8)
Diabetes mellitus	11 (1.0)	6 (0.8)	2 (1.3)	3 (1.3)
Chronic liver disease	1 (0.1)	1 (0.1)	0 (0)	0 (0)
Cancer	15 (1.3)	8 (1.1)	5 (3.2)	2 (0.8)
Immunocompromized	14 (1.3)	6 (0.8)	5 (3.2)	3 (1.3)
Other	149 (13.3)	96 (13.3)	26 (16.6)	27 (11.3)

The respective participation rate in the ensembles was on average overall 28.8% (range 19–70%), 35.7% for musicians (orchestra and choir) and 16.9% for controls. The duration of individual study participation was a maximum of 38 weeks and an average of 30 weeks. The response rate of the weekly questionnaires decreased somewhat over the entire study period and was between 67 and 99%. The response mean over the entire survey period was 80%.

### The exposure and private risk score

**Fig. 1:**
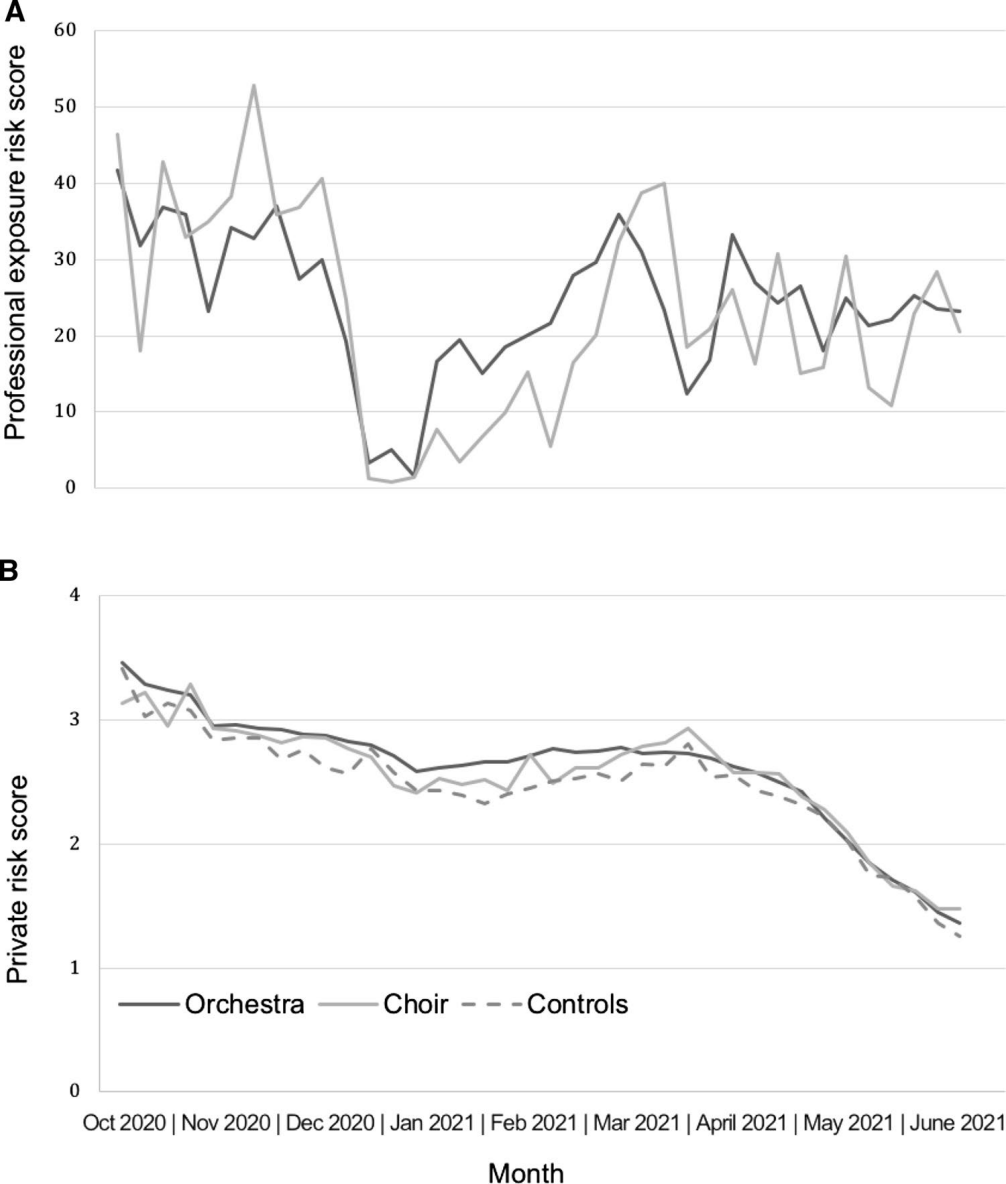
**A** Professional exposure risk score in the exposed groups orchestra and choir. **B** Private risk score in all three study groups orchestra, choir, and controls

The professional exposure risk score varied over the observation period depending on the professional activity, and the two musically active study groups orchestra and choir showed similar patterns in this respect (Fig. 1A). The private risk of infection decreased gradually over the observation period and substantially from April 2021 presumably associated with increasing vaccination rates (Fig. 1B). The three study groups orchestra, choir and controls showed similar patterns in this respect.

### Incidence of SARS-CoV-2 infection

**Fig. 2:**
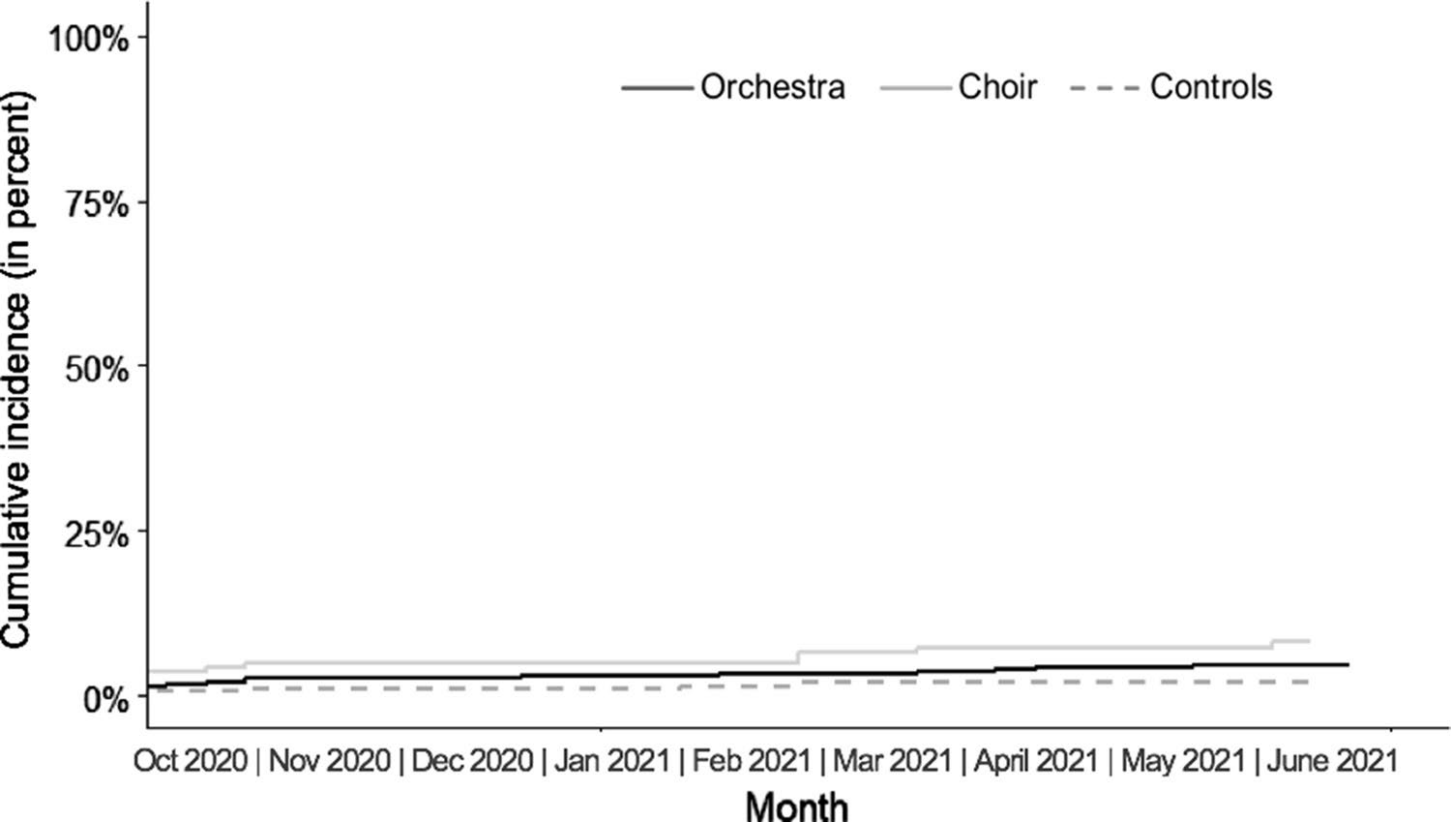
Cumulative incidence of SARS-CoV-2 infections per study group

During the observation period, 40 of 1,097 study participants tested positive for SARS-CoV-2 infection, including 26 orchestral musicians, 10 choral singers, and 4 control subjects (Fig. 2). The average weekly incidences/100.000 during the study period were: total 120.7, orchestra 119.4, choir 216.6, control 50.7. During this time, the incidence of SARS-CoV-2 infection in the German normal population was 72.4/100.000 inhabitants as calculated by us based on data from the Robert Koch Institute [25]. Taking into account the respective number of participants and the individual observation periods, the cases per person-years at risk were calculated. Hazard ratios compared to controls were 1.74 for orchestra musicians and 2.97 for choir singers (Table 2).

**Table 2:** SARS-CoV-2 Incidence in the total study cohort and in the three study groups orchestra, choir and controls; hazard ratios adjusted for mean private risk score

	Total N = 1,097	Orchestra n = 705	Choir n = 154	Controls n = 238
SARS-CoV-2 positive cases	40	26	10	4
Weeks (years) under risk	33,859 (651)	21,993 (423)	4,766 (92)	7,100 (137)
Cases per person years	0.06	0.06	0.11	0.03
Number of person years per case	16.3	16.3	9.2	34.1
Hazard ratio	–	1.74	2.97	Ref
95% confidence interval	–	0.58 to 5.25	0.87 to 10.28	–
p-value*	–	0.320	0.087	–

*Compared to controls

A comparison of orchestral musicians and choir singers with controls stratified by high and low exposure intensity showed slightly more cases per person-year in the high compared to low exposure orchestra musician group and slightly fewer cases per person-year in the high compared to low exposure choir singer group (supplementary material table S2).

Among the 40 study participants who tested positive for SARS-CoV-2, 19 suspected the source of infection in their personal environment, 5 in their professional environment, 7 expressed no suspicion, and for 9 no information was available. Of the study participants who were followed-up, 6% had no symptoms, 81% reported symptoms with no need for medical treatment, and 13% received medical outpatient treatment. The course of illness by those affected was described as mild to moderate. In the 9 participants who were not followed up, a severe course could be excluded on the basis of the information from the subsequent weekly questionnaires.

### Sensitivity analyses

The sensitivity analysis which complemented the private risk score with information from the follow-up of positive cases and the sensitivity analysis which only included contact risk yielded similar hazard ratios for orchestra musicians compared to controls: 1.73 (95% CI 0.56 to 5.35) and 2.02 (95% CI 0.70 to 5.80). Hazard ratios for choir singers compared to controls were lowest when private risk score was separated into general and contact risk (2.34, 95% CI 0.67 to 8.12) and highest when private risk score included general risk only (3.56, 95% CI 1.03 to 12.34) (supplementary material table S3).

### Incidence of influenza, flu, or upper respiratory tract infection

**Fig. 3:**
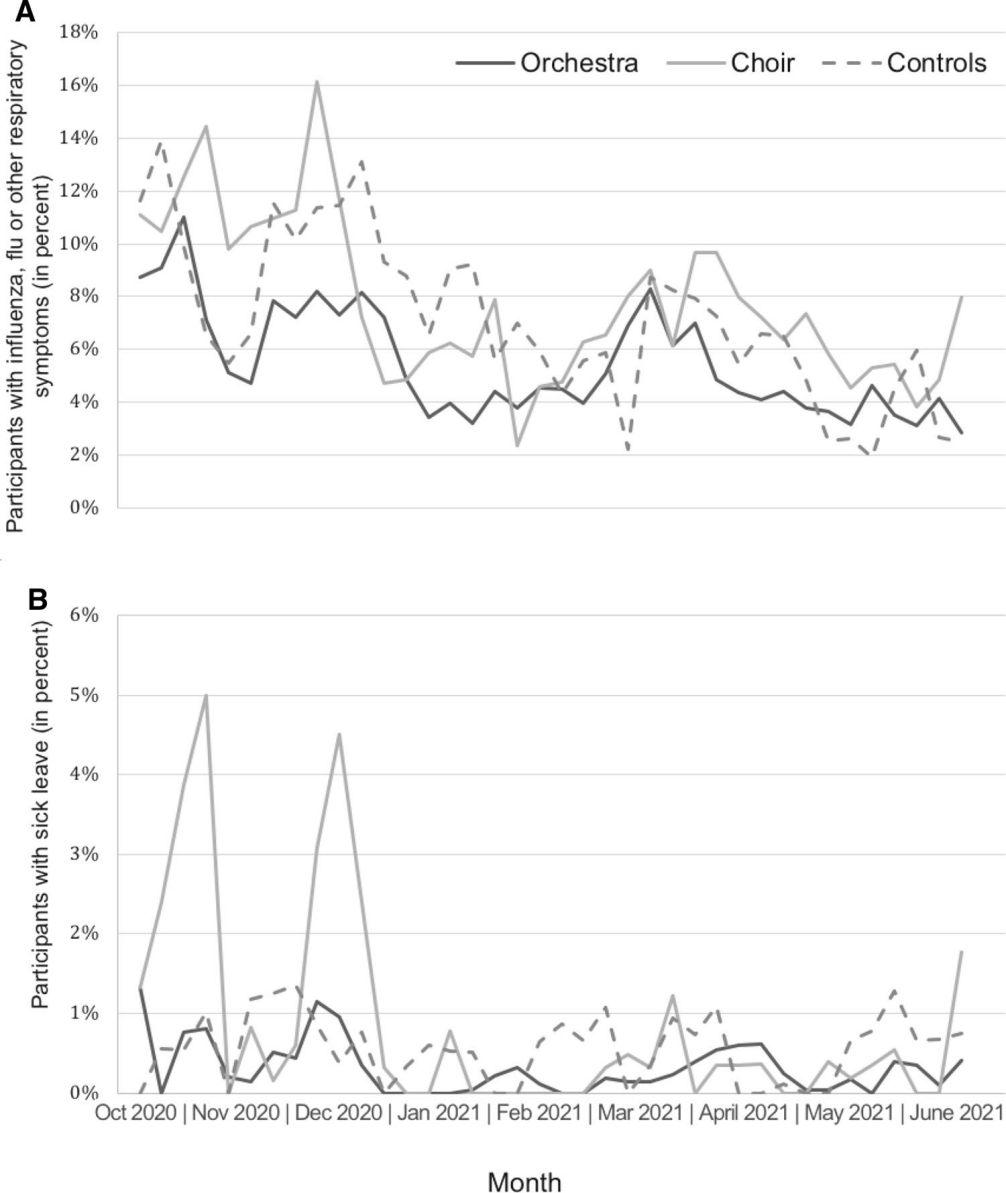
**A** Proportion of participants with influenza, flu or other respiratory symptoms per week. **B** Proportion of participants with sick leave per week

On average, participants reported cold symptoms, symptoms of influenza, or other respiratory illnesses in 7% of the study weeks; orchestral musicians tended to report less disorders than choral singers and controls (Fig. 3A). The adjusted means were 6.1% for orchestra musicians (95% CI: 4.9% to 7.3%, p = 0.085 vs. control), 10.1% for choir singers (95% CI: 7.8% to 12.3%, *p* = 0.158 vs. controls), and 8.0% for controls (95% CI: 6.1% to 9.9%).

### Number of sick leave days

On average, participants reported sick leave in 0.8% of the total study days with a tendency of fewer orchestra musicians affected than choir singers and controls (Fig. 3B). The adjusted means were 0.5% for orchestra musicians (95% CI: -0.5% to 1.4%, *p* = 0.245 vs. control), 2.1% for choir singers (95% CI: 0.7% to 3.4%, *p* = 0.411 vs. controls), and 1.3% for controls (95% CI: 0.3% to 2.4%).

## Discussion

This epidemiologic study was the first worldwide to determine SARS-CoV-2 incidence in professional orchestral musicians and choral singers compared to controls under real pandemic conditions. The professional and private risks under varying social, professional and private pandemic conditions during the entire 2020/21 performance season were continuously observed and recorded. The study period also included periods with particularly high pandemic infection numbers in Germany [25]. Throughout the study period, 40 SARS-CoV-2 positive cases occurred in the cohort. Cases per person-year under observation were 0.06 for orchestral musicians and 0.11 for choir singers versus 0.03 for controls, however, due to the low infection numbers and limited sample size these relative differences probably reflect chance. Cases occurred in different ensembles and were distributed over the observation period without any clusters. This applies only to this cohort, not to all members of all ensembles and, therefore, this study does not show representative results for any ensemble. For example, at the Bavarian State Opera, there were 61 infected persons with some clusters [16].

The intensity of exposure varied widely over the study period (e.g. due to an officially mandated lockdown, rehearsal periods, or tours), and protective measures increased over time (e.g. testing and vaccination). The minimum seat spacing for ensembles was mostly 1.5 m in all directions. Some ensembles established specific minimum distances for certain instrument and vocal groups: for wind instruments, for example, 2 to 3 m in the blowing direction or in all directions. For flutes, additional protection measures were described in three orchestras. For singers and during scenic rehearsals, depending on the ensemble, between 3 and 6 m in singing direction and 2 to 3 m in all directions were required. In individual cases, partitions made of glass, plastic or foil were also used. Special attention was paid to the ventilation systems in the rehearsal and performance rooms. As far as possible, a changeover from recirculation to fresh air ventilation in rehearsal or event rooms was made possible (n = 13) and the amount of fresh air supply was increased. Numerous ensembles used a monitoring of the aerosol concentration or the fresh air supply by a CO2 measurement. The guideline value of the CO2 concentration in the room air was 800 ppm (n = 9), the maximum value at which a sample is stopped was 1000 ppm (n = 7) or 800 ppm (n = 1). However, working conditions and hygiene concepts were subject to considerable fluctuations during the study period. In January 2021—during the third wave of the pandemic—13 ensembles reported not rehearsing, whereas 8 ensembles continued with rehearsal and online concert activities. The 13 ensembles resumed activity between February and April 2021. Audience and live concert operations were suspended entirely for several months. The return to normal activity was accompanied by different testing concepts in the ensembles (rapid antigen tests or PCR tests 2 times or more per week). All these variations in the extent of exposure were accounted for in the study methodology by combining varying individual and also ensemble-related activity into a professional exposure risk score. This approach allowed stratification according to high and low exposure.

The multiple variables influencing infection risk associated with non-occupational behavior and contacts were combined into a private risk score, for which adjustments were performed in all analyses.

The survey was conducted throughout Germany in eight (out of total 16) federal states and 11 regions. Thus, the results can be considered regionally representative for Germany and can also be applied to ensembles that did not participate in the study. Furthermore, the results may also be applicable to other performing arts sectors such as theatre and musical ensembles, where working conditions are similar to those in orchestras and choirs.

The response rate in the ensembles corresponded to the usual pattern for epidemiological studies; over the observation period participation decreased somewhat overall. The ensembles differed markedly regarding the respective proportion of participants taking part (between 19 and 70%). However, in the ensembles with a lower overall proportion of participants, they also represented the usual composition of an orchestra or choir, i.e. participants had been recruited from all instrumental and vocal groups and a relevant bias would appear unlikely.

Private infection risks that act as confounders in our study are generally difficult to ascertain and quantify. For example, the impact of family members with increased contact with SARS-CoV-2 positive individuals can vary widely. When partners work in the health care industry, they are often subject to significant hygiene and safety regimens. School children had highly variable contacts as a result of multiple periods of school closures. The impact of public transport use is also difficult to quantify. In addition, it is difficult to ascertain whether the SARS-CoV-2 positive individual recorded as a contact risk infected the study participant, making contact risk a confounder, or whether the infection took place in the reverse direction, making the contact risk a causal mediator.

In the follow-up of the positive cases in the study, private sources of infection were suspected by the persons concerned in most cases. In the case of professional infection sources suspected in the workplace, violations of hygiene concepts were blamed.

In times of pandemics, it would be very helpful to also perform similar cohort studies in other occupational groups, in order to allow for better evidence-based public health policy decisions on societal restrictions.

## Conclusion

In summary, this first epidemiologic study under real pandemic conditions in professional musicians demonstrated only few SARS-CoV-2 infections occurring among the study participants with similar incidence in orchestras versus controls, and a trend toward higher incidence in choirs versus controls. However, the exact routes of infection could not be validated in the context of this study or could not be fully clarified during a period of high incidence rates in the population. If appropriate hygiene concepts are adhered to, safe music rehearsal and performance activity appears possible even in pandemic times.

## Supplementary Information

Below is the link to the electronic supplementary material.

Supplementary file1 (DOCX 95 KB)
