# Supplementary material for: SARS-CoV-2 infections in professional orchestra and choir musicians—a prospective cohort study
Source: Eur J Epidemiol. 2022 Sep 29;37(10):1061–70. doi: 10.1007/s10654-022-00917-x (PMC9519404; doi:10.1007/s10654-022-00917-x)
Supplement: Supplementary file 1 — Supplementary file1 (DOCX 95 KB) [file 10654_2022_917_MOESM1_ESM.docx]

Supplementary Material

Supplement to Berghöfer et al SARS-CoV-2 infections in professional orchestra and choir musicians – a prospective cohort study

# Table S1: Composition of exposition score and private risk score

|  | Exposition score | Private risk score |
| --- | --- | --- |
| Items from baseline-Questionnaire |  |  |
| single person household (1) |  |  |
| multi-person household |  | 1 |
| child in household  (1, 2) |  | 1 |
| Vaccination against influenza or pneumococcus | -1 | -1 |
| Items from weekly follow-up questionnaires |  |  |
| Orchestra/choir tour in last 7 days | 1 |  |
| Teaching in the last 7 days |  |  |
| General protective measures in last 7 days |  |  |
| - If none at all | 3 |  |
| - If no mouth-nose-protection (3) |  |  |
| - If no regular airing of rooms (4) | 1 |  |
| - If no fresh air condition and ventilation system (4) | 1 |  |
| - If no other protective measures (i.e. rapid antigene or PCR testing, CO_2_-concentration measurement, etc.) | 1 |  |
| Personal protective measures in last 7 days |  |  |
| - If none at all | 4 |  |
| - If no glass partitions (5) |  |  |
| - If strings nearer than 1 m to neighbor (6) | 1 |  |
| - If wind and brass nearer than 1.5 m to neighbor | 1 |  |
| - If flutes nearer than 2 m to neighbor | 1 |  |
| - If singers nearer than 2 m to neighbor | 1 |  |
| - If controls nearer than 1 m to colleagues | 1 |  |
| - If not working in small, stable teams | 1 |  |
| - If not swabbing condensed water from wind/brass instruments(5) | 1 |  |
| - If no other personal protective measures | 1 |  |
| Risks from public |  |  |
| - Using public transport (7) |  | 1 |
| Adherence to officially ordered protection measures in daily life |  |  |
| - Most of the time |  | 1 |
| - Sometimes |  | 2 |
| - Never |  | 3 |
| Contact to groups of people |  |  |
| - Family Get-together |  | 2 |
| - Visiting a club (8) |  | 4 |
| - Visiting a restaurant (9) |  | 1 |
| - Other contact to groups of people |  | 1 |
| Contact to a person with high COVID-19 (SARS-CoV-2) spreading risk |  |  |
| - Person working in health care (10) |  | 1 |
| - Child carer or teacher (11) |  | 1 |
| - Other person with high spreading risk |  | 1 |
| Contact to a person currently SARS-CoV-2 positive *(contact risk)* |  |  |
| - with colleague from same instrument or voice group | 3 |  |
| - with other orchestra or choir colleague | 2 |  |
| - with other colleague, often | 3 |  |
| - with other colleague, seldom | 1 |  |
| - with person in my own household |  | 5 |
| - with neighbor or friend |  | 3 |
| - with other person not mentioned above |  | 1 |
| - warning in Corona-app (contact to unknown person tested positive) |  | 1 |
| Vaccination against influenza or pneumococcus (12) | -1 | -1 |
| +1 added to all subjects, to ensure min score is 0 | 1 | 1 |
| First vaccination against SARS-CoV-2 at least 2 weeks ago (13) | total weekly score*0.35 | total weekly score*0.35 |
| Second vaccination against SARS-CoV-2 at least 2 weeks ago (only first OR second vaccination factor applied, never both)  (13) | total weekly score*0.1 | total weekly score*0.1 |
| Maximum achievable (subtotal) | **18** | **28** |
| Exposition score is multiplied by rehearsal/concert time | total score * rehearsal/ concert time | **..** |
| Maximum achievable | **18 * 40 hours = 720** | **28** |

# Table S2: SARS-CoV-2 incidence in the orchestra, choir, and control group, stratified for high and low exposure intensity, hazard ratios adjusted for mean private risk score

|  | **Orchestra musician with high exposition**  **n = 353** | **Orchestra musician with low exposition**  **n = 352** | **Choir with high exposition**  **n = 77** | **Choir with low exposition**  **n = 77** | **Controls**  **n = 238** |
| --- | --- | --- | --- | --- | --- |
| SARS-CoV-2 positive cases | 15 | 11 | 4 | 6 | 4 |
| Weeks (years) under risk | 10,694 (206) | 11,299 (217) | 2,447 (47) | 2,319 (45) | 7,100 (137) |
| Cases per person years | 0.07 | 0.05 | 0.09 | 0.13 | 0.03 |
| Number of person years per case | 13.7 | 19.8 | 11.8 | 7.4 | 34.1 |
| Hazard ratio | 1.71 | 1.80 | 2.40 | 3.55 | Ref |
| 95% confidence interval | 0.54 to 5.36 | 0.55 to 5.85 | 0.56 to 10.22 | 0.91 to 13.88 | .. |
| p-value* | 0.360 | 0.330 | 0.240 | 0.069 | .. |

* compared to control group

# Table S3: Sensitivity analyses of SARS-CoV-2 incidence in the three study groups orchestra, choir and controls

|  | Orchestra  n = 705 | Choir  n = 154 | Controls  n = 238 |
| --- | --- | --- | --- |
| Private risk score updated from individual follow-up interview information |  |  |  |
| Hazard ratio | 1.73 | 3.56 | Ref |
| 95% confidence interval | 0.56 to 5.35 | 1⋅01 to 12.61 | .. |
| p-value* | 0.350 | 0.049 | .. |
| Private risk score defined by general risk only |  |  |  |
| Hazard ratio | 1.88 | 3.56 | Ref |
| 95% confidence interval | 0.61 to 5.71 | 1.03 to 12⋅34 | .. |
| p-value* | 0.270 | 0⋅045 | .. |
| Private risk score defined by contact risk only |  |  |  |
| Hazard ratio | 2.02 | 2⋅46 | Ref |
| 95% confidence interval | 0.70 to 5.80 | 0.71 to 8.50 | .. |
| p-value* | 0.190 | 0.160 | .. |
| Private risk score defined by general and contact risk separately |  |  |  |
| Hazard ratio | 1.89 | 2.34 | Ref |
| 95% confidence interval | 0.66 to 5.45 | 0.67 to 8.12 | .. |
| p-value* | 0.240 | 0.180 | .. |

* versus controls

1. Lei H, Xu X, Xiao S, Wu X, Shu Y. Household transmission of COVID-19-a systematic review and meta-analysis. Journal of Infection. 2020;81(6):979-97. doi:10.1016/j.jinf.2020.08.033

2. Ehrhardt J EA, Krehl H, Meincke M, Finci I, Klein J, Geisel B, Wagner-Wiening C, Eichner M, Brockmann SO. Transmission of SARS-CoV-2 in children aged 0 to 19 years in childcare facilities and schools after their reopening in May 2020, Baden-Württemberg, Germany. Euro Surveill. 2020. doi:<https://doi.org/10.2807/1560-7917.ES.2020.25.36.2001587>

3. Tabatabaeizadeh S-A. Airborne transmission of COVID-19 and the role of face mask to prevent it: a systematic review and meta-analysis. European Journal of Medical Research. 2021;26(1):1. doi:10.1186/s40001-020-00475-6

4. Morawska L, Tang JW, Bahnfleth W, et al. How can airborne transmission of COVID-19 indoors be minimised? Environ Int. 2020;142:105832. doi:<https://doi.org/10.1016/j.envint.2020.105832>

5. Firle C, Jabusch H, Grell A, Fernholz I, Schmidt A, Steinmetz A. Musizieren während der SARS-CoV-2-Pandemie. Empfehlungen der Deutschen Gesellschaft für Musikphysiologie und Musikermedizin (DGfMM) zum Infektionsschutz beim Musizieren Hamburg: Deutsche Gesellschaft für Musikphysiologie und Musikermedizin (DGfMM)2020 13.8.2020.

6. Chu DK, Akl EA, Duda S, et al. Physical distancing, face masks, and eye protection to prevent person-to-person transmission of SARS-CoV-2 and COVID-19: a systematic review and meta-analysis. The Lancet. 2020;395(10242):1973-87. doi:10.1016/s0140-6736(20)31142-9

7. Hu M, Lin H, Wang J, et al. Risk of Coronavirus Disease 2019 Transmission in Train Passengers: an Epidemiological and Modeling Study. Clin Infect Dis. 2021;72(4):604-10. doi:10.1093/cid/ciaa1057

8. Muller N KM, Steitz F, Saad NJ, Mühlemann B, Beheim-Schwarzbach JI, et al. Severe Acute Respiratory Syndrome Coronavirus 2 Outbreak Related to a Nightclub, Germany, 2020. Emerg Infect Dis. 2020;2021;27(2):645-648. doi:10.3201/eid2702.204443

9. Fisher KA TM, Feldstein LR, et al. Community and Close Contact Exposures Associated with COVID-19 Among Symptomatic Adults ≥18 Years in 11 Outpatient Health Care Facilities — United States, July 2020. . MMWR Morb Mortal Wkly Rep 2020;69:1258–1264. 2020. doi:10.15585/mmwr.mm6936a5external icon

10. Gómez-Ochoa SA, Franco OH, Rojas LZ, et al. COVID-19 in Health-Care Workers: A Living Systematic Review and Meta-Analysis of Prevalence, Risk Factors, Clinical Characteristics, and Outcomes. Am J Epidemiol. 2021;190(1):161-75. doi:10.1093/aje/kwaa191

11. Thomas G. Grobe SB, Bertolt Meyer, Alexander Zill, Susen Schuhmann, Sai-Lila Rees, Hannah C. Tendyck , Albrecht Wehner, Micaela Berger, Jenny Wirth, Stefan Mortz, Andreas Volkmar. Dossier 2020 – Corona 2020: Gesundheit, Belastungen, Möglichkeiten. Techniker Krankenkasse, Hamburg. 2020.

12. Jehi L, Ji X, Milinovich A, et al. Individualizing Risk Prediction for Positive Coronavirus Disease 2019 Testing: Results From 11,672 Patients. Chest. 2020;158(4):1364-75. doi:10.1016/j.chest.2020.05.580

13. Harder T KJ, Vygen-Bonnet S, Scholz S, Pilic A, Reda S, Wichmann O. Wie gut schützt die COVID-19-Impfung vor SARS-CoV-2-Infektionen und SARS-CoV-2-Transmission? – Systematischer Review und Evidenzsynthese. Epid Bull. 2021;19/2021:13 -23. doi:10.25646/8442
